# Topics of Public Interest

**Published:** 1914-09

**Authors:** 


### TOPICS OF PUBLIC INTEREST.

---

**The Pre-marital Medical Certificate Law** has been declared constitutional by the Supreme Court of Wisconsin, reversing the decision of a lower court.

---

**Independence Day Casualties** for this year are reported to be 13 deaths and 243 injuries as against 32 and 1,121 respectively last year.

---

**Stirpiculture and Early Marriage.** Casper L. Redfield of Chicago in a pamphlet, casts doubt on the desirability of early marriage. He alludes to Roswell H. Johnson's article in the Journal of Heredity, Vol. 5, page 102 in which it is shown that of two stocks, one having 4, the other 3 generations to a century, the latter will constitute only a third of the total at the end of a century, on the basis of 4 surviving children to each marriage.

Mr. Redfield has compiled a table from various American Genealogies, giving the ages of grandfathers at the time the first grandchild was born and the total number of grandchildren in each instance. From 1 grandchild to men first becoming grandfathers at the ages of 40 and 41, the number gradually increases to 33 at 48; 40 at 50; averages 75 for the ages 54 to 57; reaches the maximum of 104 at the age of 64; declines to 73 for the age 72 and then declines gradually but we find 9 grandsons to men 100 and over when their first grandchild was born. This table would be more convincing if, instead of the aggregate number of grandsons for a group of grandfathers of a given age when the first grandson was born, we knew the average at each age. In its present form, it simply shows what has resulted under ordinary social conditions.

However, Mr. Redfield backs up his claim with the following offers: $100 for the demonstration that any of the 2,000 or 3,000 intellectually eminent men known to history come within the 4 generation to the century class or, as an alternate $100 for the demonstration that more than 3 of this group come in this class for the male line alone; $100 if any of the exceptionally eminent men can be found in the 3 generation to the century class. These two prizes will be given to the American Genetic Association, not to the prize winner.

---

**New Way of Victimizing the Affected.** Louis H. Ward, Attorney of the California State Board of Medical Examiners has found in the offices of certain quacks, interleaved copies of Parke, Davis & Co.'s price list, with fake specialties listed at exorbitant rates. The special leaves, of which fac-similes are given by Mr. Ward, follow closely the type and methods of description of the genuine leaves and contain such gems as the following: Lymph Compound. Absolute specific for Sexual Neurasthenia, Nervous Collapse, Debilitated Condition and Brain Disorders. The prices range from $5.25 for 2000 Units up to $43 for 10,000 Units. Cat Serum, tubes 1, 2 and 3, is priced at $20.45. $21.60 and $26.40 respectively. Rectal suppositories containing ext. cannabis indica, ext. berberine, ext. witch hazel, boracic acid, glycerine and cocoa butter are listed at $21.36 per dozen. Several other formulae at corresponding prices are quoted and in prominent type are reiterated such statements as "Sold to physicians only," "Draft or money order must accompany orders." "Prices quoted are net," etc. The obvious intention of the interleaves is to be able to show the patient, in the price list of a well known firm, an excuse for an excessive charge for medical treatment. Messrs. Parke, Davis & Co. fail to appreciate the compliment paid them and while, under the circumstances, a disclaimer from them is unnecessary, they wish to aid in every way in suppressing this new way of imposing upon a credulous public.

---

**Otophone.** Dr. Fournier d'Albe, demonstrated before the Royal Society, in July, a new instrument, his own invention, by which the blind may be enabled to read through the transformation of light vibrations into those of sound. A perforated disc revolves a powerful Nernst lamp. A small, intensely bright line of light passing through these holes travels from one letter to the next of the printed or written sheet and the type reflects the light upon a selenium bridge. Each letter gives a characteristic sound, which is heard through a telephone. After learning these characteristic sounds, the blind can follow the print.

---

**The J. N. Adam Memorial Hospital,** through the Supt., Dr.

Clarence L. Hyde, makes the following announcement: All patients applying for admission, including children with tuberculosis of the bones and glands, must proceed as follows: Apply at the Dept. of Health, Church and Franklin Streets, Buffalo. Examinations will be made as to suitability of the applicant by Dr. Hyde or Dr. Lo Grasso, at the Tuberculosis Dispensary, 175 E. Swan Street, at 3:30 p.m., Mondays. Notices will be sent to applicants when to apply for examination.

---

**Federal Meat Inspection.** New regulations were put into force, July 15, applying to interstate commerce which comprises about 60% of the slaughtering of the country. Animals condemned previous to slaughter must be killed in a separate place and the products used for fertilizer only. Careful post mortem examinations will be made of all carcasses before marking them "U. S. Inspected and Passed." Inspection of houses violating any regulation will be withdrawn, so that the house cannot ship into other states or foreign countries. On account of the high price of meat products, firms will be allowed to sell as "Second class Sterilized," lard and meat from carcasses presenting only local lesions, when thoroughly sterilized by heat. Raw pork is prohibited in summer sausage or similar foods customarily used without further cooking. Various regulations are made to secure cleanliness of premises and employes, asepsis of knives, etc., and the regulations regarding imported meat have been standardized.

---

**Practical Result of Boylan Law.** The Buffalo Poor Dept. reported August 1 that 325 drug habitues were under treatment on its orders in various hospitals of the city.

---

**The Lucien Howe Prize** of the Medical Society of the State of N. Y. has been awarded to Dr. Mark J. Schoenberg for research work in ocular tuberculosis.

---

Mrs. Carrie S. Austin, a trained nurse, has been appointed as the first Policewoman of Buffalo. This journal was among the first to call attention to this subject and to advocate the appointment of policewomen in the cities of western N. Y.

---

**Convicted Drug Users Subject to Support at Public Expense.** Attorney General Carmody has ruled that as the Boylan law fails to place the burden of support upon drug users themselves or upon their relatives, they must be supported by the state, county, or city according to the institution to which they are committed.

---

**Drug Traffic in Auburn Prison.** The N. Y. State Prison

Committee, on investigation, declares that exaggerated ideas have prevailed as to the amount of drug addiction among prisoners. It gives credit to the Mutual Welfare League of the prisoners themselves.

---

**Gift to Albion Hospital.** Arnold Gregory, who donated a site and building to Albion two years ago, for hospital use, has recently given $30,000 to remodel and equip the hospital.

